# The First National Symposium on Sleep Disorders, Jeddah, Saudi Arabia: January 30–31, 2008

**Published:** 2008

**Authors:** Ayman Krayem

**Affiliations:** *Department of Medicine, Respiratory and Sleep Disorders Section, King Abdulaziz Medical City, Jeddah, Saudi Arabia*

Sleep Disorders is a rapidly growing field of medicine that has attracted much interest over the past several years owing to its significant impact on health and society. ‘The First National Symposium on Sleep Disorders’ was among the first of its kind conducted in Saudi Arabia on the 30^th^ and 31^st^ of January 2008. The symposium, targeting a wide range of health-care professionals, was designed to create increasing awareness of various sleep disorders in order to facilitate their early recognition, referrals and proper management.

The time was right to move forward in the field of ‘Sleep Medicine’ in Saudi Arabia since the number of sleep specialists has increased in the past several years. As well, the number of sleep laboratories and centers are becoming more available and are currently treating increasing number of patients diagnosed with sleep disorders.

The symposium was organized by the Sleep Disorders Center, King Abdulaziz Medical City, Jeddah, and endorsed by the American College of Chest Physicians (ACCP) and the Saudi Thoracic Society (STS). It received 10 h of continued medical education (CME) accreditation by the Saudi Council for Health Specialties (SCFHS).

This event was considered by many as a cornerstone to improve the infrastructure of sleep medicine in Saudi Arabia and help spread its knowledge among health-care providers, especially those caring for patients with high risk for sleep-disordered breathing (SDB).

The scientific committee (Dr. Siraj Wali, Dr. Ayman Krayem, Prof. Ahmed BaHammam and Dr. Charles George) faced the challenge to create a program that met the needs of all participants while still being able to cover the most important topics in the field of sleep medicine in an attractive manner.

Six sessions were arranged to cover the purpose of this symposium, which included:

1. Introduction to sleep medicine (including sleep in health and its current status in Saudi Arabia)
2. Obstructive sleep apnea syndrome
3. Sleep in pediatrics (including parasomnias)
4. The patient with excessive movement in sleep [including restless legs syndrome (RLS), parkinsonism and epilepsy]
5. The sleepy patient (including narcolepsy)
6. Miscellaneous topics in sleep medicine (including sleep in women, chronic lung diseases, allergic and psychiatric disorders)

In between sessions, two grand-round lectures were given - ‘The Sleepy Driver’ and ‘The Patient Who Cannot Sleep’. The speakers were all talented consultants in their field from different institutes in the Kingdom, caring for patients with SDB, insomnia and movement disorders; and most were Sleep Physicians managing patients with various sleep disorders and supervising sleep laboratories and centers in their hospitals. Among the speakers, the symposium hosted a guest speaker from Canada, Dr. Charles George, a known expert and author in the field of sleep medicine, especially OSA in relation to driving.

Two workshops were also conducted during the 2-day meeting. The first provided a hands-on experience with the basics of CPAP/BiPAP setup and troubleshooting in SDB. The second was to orient attendees with the basics of polysomnography (PSG) setup and their interpretations. These were closed sessions for 25 persons each, mainly physicians and respiratory therapists.

The introductory lecture by Dr. George gave an orientation of the physiology of normal sleep in healthy adults. The question ‘why we sleep’ was addressed, and the importance of good and enough sleep was highlighted. This was followed by two lectures given by Prof. Omer Al-Amoudi and Dr. Hamdan AlJahdali highlighting the approach to patients with sleep disorders in general, and they gave an overview of the sleep disorders classification as per the International Classification of Sleep Disorders (ICSD). In summary, in the approach for diagnosing OSA, it was recommended to include the following:

1. Patients that present with loud snoring and excessive daytime sleepiness (EDS) should undergo polysomnography (PSG).
2. In the absence of EDS, PSG may be performed if two or more of the following are present: witnessed apneas, awakening with choking, nocturnal restlessness, change in mood, morning headaches, GERD, obesity, essential hypertension, arrhythmias, cor pulmonale, polycythemia and unexplained pulmonary hypertension.
3. In the absence of clinical features of OSA, PSG may be performed only on patients with mission-critical professions (e.g., airline pilots, bus drivers).
4. Negative PSG should be viewed with skepticism if the clinical suspicion of OSA is high; therefore, a repeat PSG should be considered, or preferably patients should be followed clinically.

Sleep medicine in Saudi Arabia, its present and future were reviewed in a subsequent presentation. Prof. Ahmed BaHammam reviewed recent papers addressing physician awareness of sleep disorders and highlighted the literature that points out the negative impact of insufficient sleep disorders, facilities capable of diagnosing and treating different sleep disorders in KSA. Regrettably, this service is still considered underdeveloped and underutilized, and many obstacles are faced by sleep medicine practitioners in Saudi Arabia.

The series of presentations then focused on the major topic of OSA, which attracted most of the audience attention. A general overview of OSA followed by consequences of untreated OSA in terms of cardiovascular and cerebrovascular events, together with CPAP treatment efficacy, was the main theme of this session. Dr. Ayman Krayem, Dr. Javed Khan and Dr. Charles George talked about OSA in terms of epidemiology, major risk factors (obesity, male gender, older age and snoring), prognosis and medical management. Untreated OSA was regarded as an established independent risk factor for the development of systemic hypertension. The audience then was engaged in a very fruitful discussion with the speakers. CPAP was presented as an important intervention that is proven to reduce the risk of CV incidents and improve mortality rate. The stage was then set to continue this session with lectures addressing treatment options for OSA, which included upper airway procedures such as UPPP, laser-assisted and tissue-volume-reduction surgery, given by Dr. Khalid Alnouri; and bariatric surgery with its various techniques and efficacy, given by Dr. Adel Bakhsh. Dr. Khalid Azzam then concluded the session on OSA by talking about dental/oral devices used as a way to manage specific cases of OSA. The role of the dentist as part of the multidisciplinary team was highlighted since the dental/oral appliances can treat mild-to-moderate snoring/sleep apnea, as a simple alternative to conventional therapies.

As part of the four major topics assigned to him, Dr. George gave a grand-round lecture on the subject of his primary interest and research work - sleepiness and driving risks. Literature about inattention as a major factor in motor vehicle accidents (MVA), especially in vigilance-sensitive occupation such as commercial and bus driving, was presented. Data about OSA and increased risks for MVA that can be reduced with proper treatment with CPAP were also reviewed.

The participants then dispersed and gathered again for the two workshops highlighted earlier. These workshops were conducted by Ms. Buthainah Alghamdi (Basics of Polysomnography), Mr. Abdulsalam Alzahrani and Mr. Foad Almutairy (CPAP/BiPAP setup). Dr. Siraj Wali supervised both the workshops, whose objectives were to:

1. Discuss the most common indications of PSG
2. Learn the basics of patients’ hookup for a PSG
3. Recognize the basic scoring rules of sleep stages and respiratory events
4. Learn when and how to apply positive pressure therapy (PPT) in SDB
5. Trouble-shoot the technical issues related to this therapy
6. Recognize the differences between the available CPAP and BiPAP machines

The second day consisted of the remaining four sessions focusing on other important topics on sleep medicine. These attracted the attention of many attendees caring for children with suspected sleep problems and movement disorders including parasomnia. These issues were well explained by Dr. Muslim Alsaadi and Dr. Fawzia Bamoqaddam. OSA is recognized as one of the most common respiratory disorders of childhood, affecting an estimated 1–2% of normal children. Its diagnosis requires special expertise since the diagnostic PSG criteria are quite different between adults and children and adolescents. Parasomnias are defined as disorders characterized by undesirable motor, verbal or autonomic nervous system changes occurring in association with sleep. They were viewed as potential cause for sleep-related injuries and psychological distress from repeated loss of self-control during sleep.

Along the same line, Dr. George and Dr. Ahmed Attar covered movement disorders in adults in the next session titled ‘The Patient with Excessive Movement in Sleep’, which reviewed idiopathic and secondary RLS. This problem occurs particularly when one lies down in bed, because rest is associated with irresistible leg movements that interfere with sleep onset. Other movement disorders particularly associated with Parkinson's disease and epilepsy were also reviewed.

The next theme “Why Am I Sleepy” discussed various causes of excessive daytime sleepiness, including narcolepsy and sleep apnea, and highlighted the health-related and social impact of sleep deprivation. A practical approach to the diagnosis of variable causes of excessive sleepiness was presented. Sleep deprivation as one of the commonest sleep disorders associated with serious consequences on many levels, including health, family, community, education and others, was particularly emphasized. These topics were covered in the two talks given by Dr. Siraj Wali and Prof. Ahmed BaHammam respectively.

Insomnia is a major topic in sleep medicine; and Dr. Ayman Krayem discussed its causes, epidemiology and management approach during the second grand-round lecture. Chronic psychophysiologic insomnia was given particular attention as it is among the most common forms of insomnia. Cognitive behavioral therapy (CBT), stimulus control and good sleep hygiene, along with targeted short-term drug therapy, were among the most successful treatment options discussed for this and other types of insomnias. Dr. Mohammed Al-Barrak, who chaired the insomnia session, then presented ‘Sleep Disorders in Women in Pre- and Postmenopausal Periods’, an issue that is under-represented in the literature. ‘Hormonal change in women’ was reviewed as a potential explanation for the gender-related differences in disorders such as sleep apnea.

As well, understanding the close relationship between sleep and chronic respiratory disorders such as bronchial asthma and COPD was an important topic covered by Dr. Saleh Al-Dammas. Nocturnal asthma and sleep hypoventilation experienced by many moderate-to-severe COPD patients were among the problems discussed. This talk was followed by a presentation about sleep and psychiatric disorders such as depression. Dr. Tariq Sherif talked about risk factors for sleep disorders associated with psychiatric disorders, which included old age, anxiety, depression, lower social status and multiple health problems.

The symposium was concluded with a presentation given by Prof. Emad Koshak regarding the common but significantly underappreciated problem of sleep and allergic disorders. Allergic disorders such as asthma, allergic rhinitis, atopic dermatitis and food allergy affect 1–2 in 10 individuals worldwide. The assessment of allergy was recommended as an integral part of management of patients with sleep disorders, particularly in patients uncontrolled on conventional therapy.

The organizing and scientific committees, together with the chair and senior members of the Saudi Thoracic Society, expressed their satisfaction with the way the symposium was conducted and hoped for organizing a subsequent larger-scale event in the field of sleep medicine with significant research orientation and contribution by many national and international speakers.

Words/Group of words/Corrections that need to be checked/verified have been highlighted or commented upon. Those abbreviations used for the first time in the article but not spelt out are highlighted; along with the abbreviations, their expanded forms need to be given at the place where the abbreviations are first used.

